# Proprotein convertase furin inhibits matrix metalloproteinase 13 in a TGFβ-dependent manner and limits osteoarthritis in mice

**DOI:** 10.1038/s41598-018-28713-2

**Published:** 2018-07-11

**Authors:** Hilène Lin, Eric Hay, Augustin Latourte, Thomas Funck-Brentano, Wafa Bouaziz, Hang-Korng Ea, Abdel-Majid Khatib, Pascal Richette, Martine Cohen-Solal

**Affiliations:** 1 INSERM 1132, USPC Université Paris-Diderot, Paris, France; 2 Department of rheumatology, Hôpital Lariboisière, Paris, France; 3 University of Bordeaux, INSERM, LAMC, UMR 1029, F-33400 Talence, France

## Abstract

Cartilage loss in osteoarthritis (OA) results from altered local production of growth factors and metalloproteases (MMPs). Furin, an enzyme involved in the protein maturation of MMPs, might regulate chondrocyte function. Here, we tested the effect of furin on chondrocyte catabolism and the development of OA. In primary chondrocytes, furin reduced the expression of MMP-13, which was reversed by treatment with the furin inhibitor α1-PDX. Furin also promoted the activation of Smad3 signaling, whereas activin receptor-like kinase 5 (ALK5) knockdown mitigated the effects of furin on MMP-13 expression. Mice underwent destabilization of the medial meniscus (DMM) to induce OA, then received furin (1 U/mice), α1-PDX (14 µg/mice) or vehicle. In mice with DMM, the OA score was lower with furin than vehicle treatment (6.42 ± 0.75 vs 9.16 ± 0.6, p < 0.01), and the number of MMP-13(+) chondrocytes was lower (4.96 ± 0.60% vs 20.96 ± 8.49%, p < 0.05). Moreover, furin prevented the increase in ALK1/ALK5 ratio in cartilage induced by OA. Conversely, α1-PDX had no effect on OA cartilage structure. These results support a protective role for furin in OA by maintaining ALK5 receptor levels and reducing MMP-13 expression. Therefore, furin might be a potential target mediating the development of OA.

## Introduction

Osteoarthritis (OA) is the most prevalent form of arthritis and a leading cause of pain and physical disability, especially in the elderly^1^. One major hallmark of the disease is the breakdown of articular cartilage matrix, especially type II collagen and proteoglycans, by several proteases^2^. Aggrecanases, such as a disintegrin and metalloproteinases with thrombospondin motifs (ADAMTS)−4 and −5, mediate cartilage remodelling^3^ and their inhibition prevents OA-associated aggrecan degradation^4^. In addition, ADAMTS-5 deficient mice are protected against OA^5^. Similarly, matrix metalloproteinase (MMP)−13 is a major collagenase responsible for the degradation of type II collagen^6^. Increased expression of MMP-13 is observed in human OA cartilage^7,8^, and its knockout protects against cartilage destruction in murine OA^9^. Both *Adamts5* and *Mmp13* are target genes for transforming growth factor beta (TGFβ) in articular chondrocytes^10^. TGFβ, which is secreted by chondrocytes and stored within the matrix plays a crucial role in cartilage homeostasis^11,12^. Indeed, disruption of TGFβ signaling results in chondrocyte hypertrophy and cartilage breakdown^13^. The effects of TGFβ in chondrocyte metabolism are driven through its binding with TGFβ type II receptor (TGFβRII), leading to the recruitment of 2 activin receptor-like kinase (ALK) receptors. ALK1 activates the Smad1/5/8 pathway thereby leading to induction of chondrocyte hypertrophy and increased catabolism. By contrast, ALK5 receptor activates Smad2/3 signaling pathway involved in chondrocyte differentiation and anabolic response^14^. In addition, deletion of TGFβRII up regulates the expression of MMP-13 and ADAMTS-5 and results in OA-like phenotype in mice^10^.

ALK1/ALK5 balance is therefore crucial for chondrocyte metabolism and cartilage maintenance. A shift from ALK5 to ALK1 signaling occurs in OA and upon ageing^14^, leading to increased MMP-13 expression^15^.

Furin convertase, an enzyme involved in the maturation of chondrocytic proteases, also regulates TGFβ activity^16^. Furin was found to promote the *in vitro* maturation of MMPs in chondrocytes^17^ and reduce collagen degradation^18^. Hence, furin colocalizes with pro-ADAMTS-4 in the trans-Golgi network, where it interacts with the pro-domain; its inhibition induced its maturation *in vitro*^19^. However, blocking furin dampened the secretion of the pro and mature forms of MMP-13 in cartilage explants (Milner) or in the presence of a latent small TGFβ1 complex^16^, which indicates that MMP-13 inhibition is mediated by maturation of TGFβ. Overall, previous reports suggesting that furin promotes cartilage breakdown via MMP activation but induces the maturation of TGFβ have been addressed by *in vitro* methodology. Therefore, knowledge of the integration of the different targets of furin in cartilage and OA conditions is needed. Here, we investigated the impact of furin on TGFβ-induced MMP-13 activation in chondrocytes, and assessed its effect in an experimental model of murine OA.

## Results

### Furin reduces MMP-13 expression in primary chondrocytes

**Figure 1:**
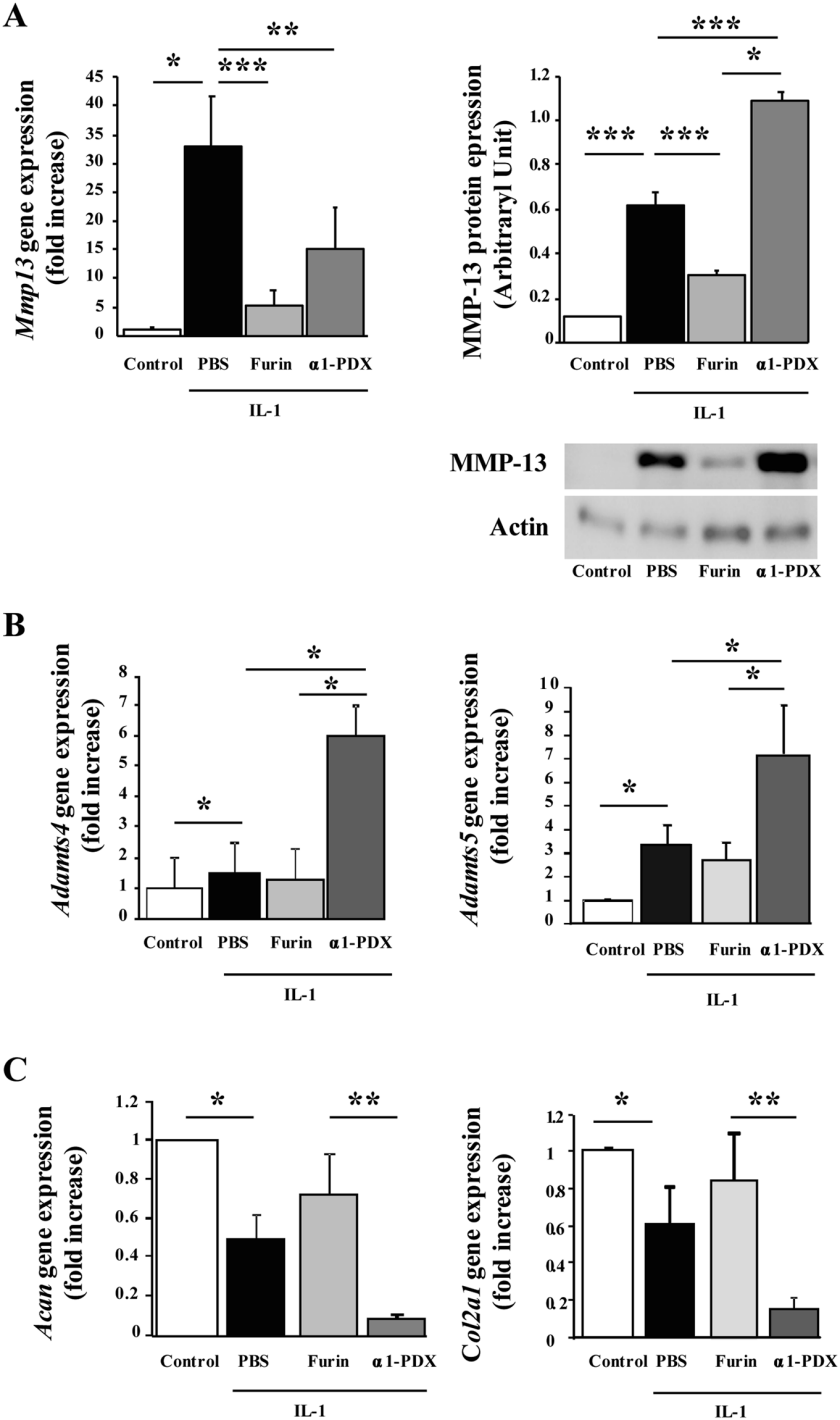
Furin regulates MMP-13 activation in IL-1-stimulated chondrocytes. Primary mouse chondrocytes were stimulated with IL-1 and then cultured with PBS, Furin, α1-PDX. (**A**) Quantitative RT-PCR and western blot analysis of mRNA and protein expression of MMP-13, in controls or interleukin-1 (IL-1)-stimulated primary chondrocytes with PBS, Furin (10 U/mL) and α1-PDX (8 µM) treatment. Data for RT-PCR are fold increase relative to control and signal intensity relative to Actin for western blot. (**B**) Quantititative RT-PCR analysis of mRNA levels of ADAMTS-4 and ADAMTS-5 expressed as fold increase. (**C**) Quantititative RT-PCR analysis of mRNA levels for aggrecan (*Acan*) and type II collagen (*Col2a1*). Data are mean±SEM. *p < 0.05, **p < 0.01, ***p < 0.001.

To test the effect of furin on proteases expression, murine chondrocytes were pre-stimulated with interleukin (IL)−1 and then cultured with PBS or furin or its inhibitor α1-PDX. Furin markedly reduced the IL-1-induced *Mmp13* mRNA expression (5.23 ± 2.70 vs PBS: 32.87 ± 8.84-fold increase, p < 0.001). This inhibition was lower with α1-PDX (14.97 ± 7.27-fold increase, p < 0.01, Fig. 1A). Such profile was confirmed at the protein level. Furin significantly blunted the IL-1 induced protein expression of MMP-13 (0.30 ± 0.02 vs. 0.62 ± 0.05 AU, p < 0.0001). Conversely, α1-PDX even increased the MMP-13 expression upon IL-1 treatment (1.09 ± 0.04, p < 0.001), suggesting that endogen furin limits the production of MMP-13 in IL-1-stimulated chondrocytes by regulating posttranslational processes (Fig. 1A). Furin did not significantly change the mRNA expression of *Adamts4* and *Adamts5*, but these levels were enhanced with α1-PDX, which suggests the protective role of furin in the presence of IL-1 (Fig. 1B).

By contrast to the effects observed with metalloproteases, IL-1 significantly lowered the mRNA expression of anabolic genes *Acan* (aggrecan) and *Col2a1* (type II collagen) (0.49 ± 0.14 and 0.63 ± 0.12, p < 0.05). Furin failed to restore the expression of *Acan* or *Col2a1* (0.726 ± 0.214 and 0.847 ± 0.247, p = 0.07 and p = 0.09, respectively), whereas α1-PDX further aggravated the effects of IL-1 on these genes (0.08 ± 0.001- and 0.16 ± 0.04-fold increase, p < 0.01, Fig. 1C).

### Furin activates ALK5/Smad3 signaling

**Figure 2:**
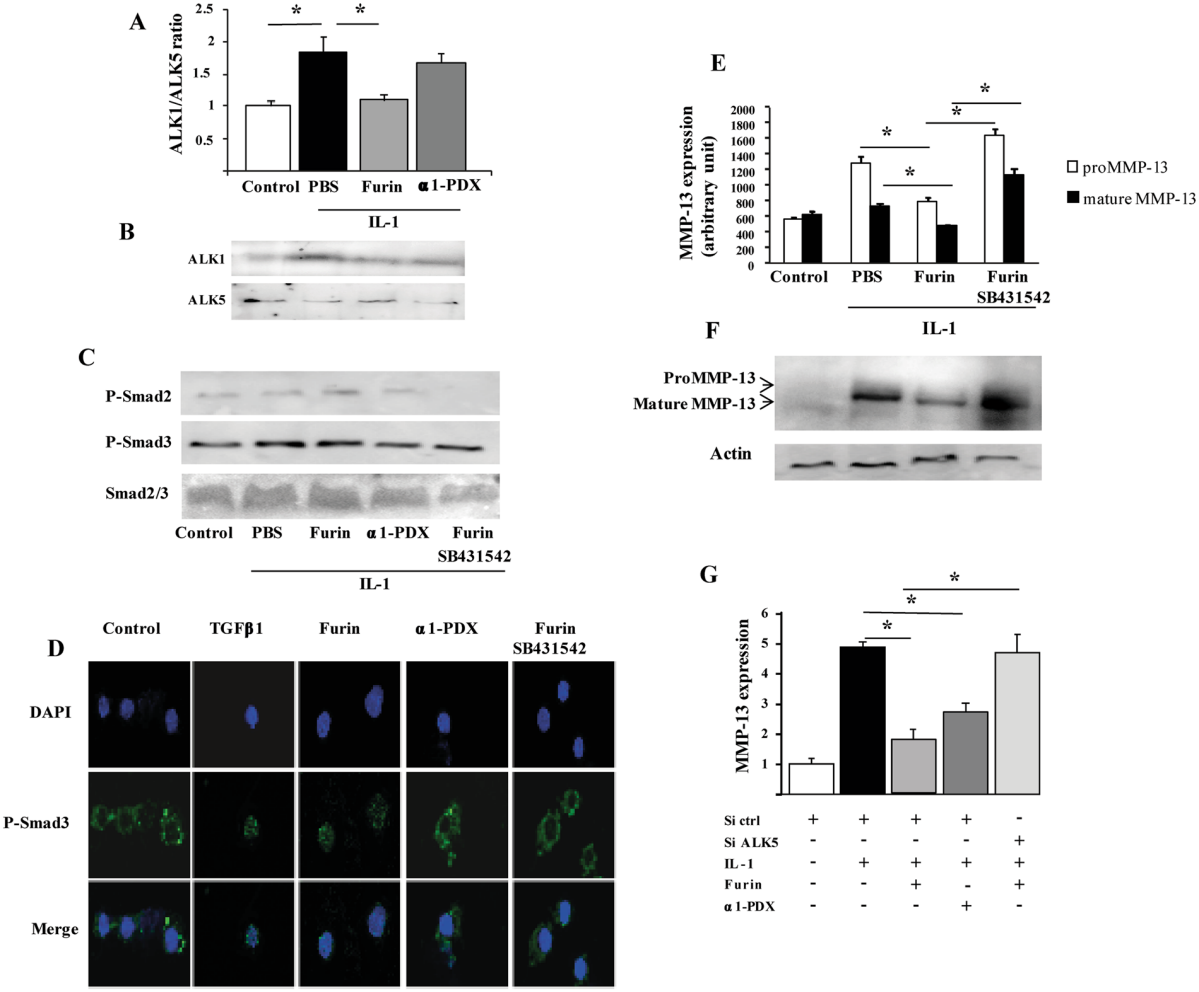
Furin activates ALK5/Smad3 signaling and reduces MMP-13 expression in primary chondrocytes. Primary chondrocytes were incubated with IL-1, and then cultured with PBS, Furin, α1-PDX and Furin plus TGFβ1/ALK5 inhibitor SB431542. (**A**) Quantification of ALK1/ALK5 ratio (arbitraty units) as detected by Western blot. (**B**) Representative images of Western blot analysis of ALK1 and ALK5. (**C**) Western blot analysis of phosphorylation of Smad2, Smad3 and Smad2/3. (**D**) Immunocytochemistry of p-Smad3 translocation (X600). (**E**) Quantification of MMP-13 protein expression (proform and mature form) as detected by Western blot, actin being the loading control. (**F**) Representative images of Western blot analysis of pro and mature MMP-13 protein. (**G**) Quantitative RT-PCR analysis of MMP-13 mRNA expression in chondrocytes with or without *Alk5* siRNA knockdown. Results are expressed as fold increase relative to controls. Data are mean±SEM. *p < 0.05.

Because MMP-13 is a target of TGFβ signalling, we investigated whether the downregulation of MMP-13 induced by furin was mediated by TGFβ pathway. In IL-1-stimulated chondrocytes, the ratio of ALK1/ALK5 increased compared to controls (1.8 ± 0.27 fold, p < 0.05). Furin restored an ALK1/ALK5 ratio similar to that of controls by reducing the expression of ALK1 and reversing the inhibition of ALK5 (Fig. 2A,B). Additionally, furin promoted the phosphorylation of Smad3 (Fig. 2C) and the translocation of P-Smad3 into the nucleus (Fig. 2D). These effects were reversed by the TGFβ/ALK5 inhibitor SB431542. Interestingly, the protective effect of furin on the production of both proform and mature form of MMP-13 in IL-1-stimulated chondrocytes was lost in presence of SB431542 (Fig. 2E,F). We then knocked down ALK5 by siRNA which allowed a 90% reduction of *Alk5* gene expression (not shown). In cells transfected with siALK5, furin had no effect on IL-1-induced MMP-13 expression (Fig. 2E). Overall, these findings demonstrate that ALK5/Smad3 pathway mediates the protective effects of furin on MMP-13 production induced by IL-1 in chondrocytes.

### Furin reduces OA cartilage lesions and modulates ALK1/ALK5 ratio in mice

**Figure 3:**
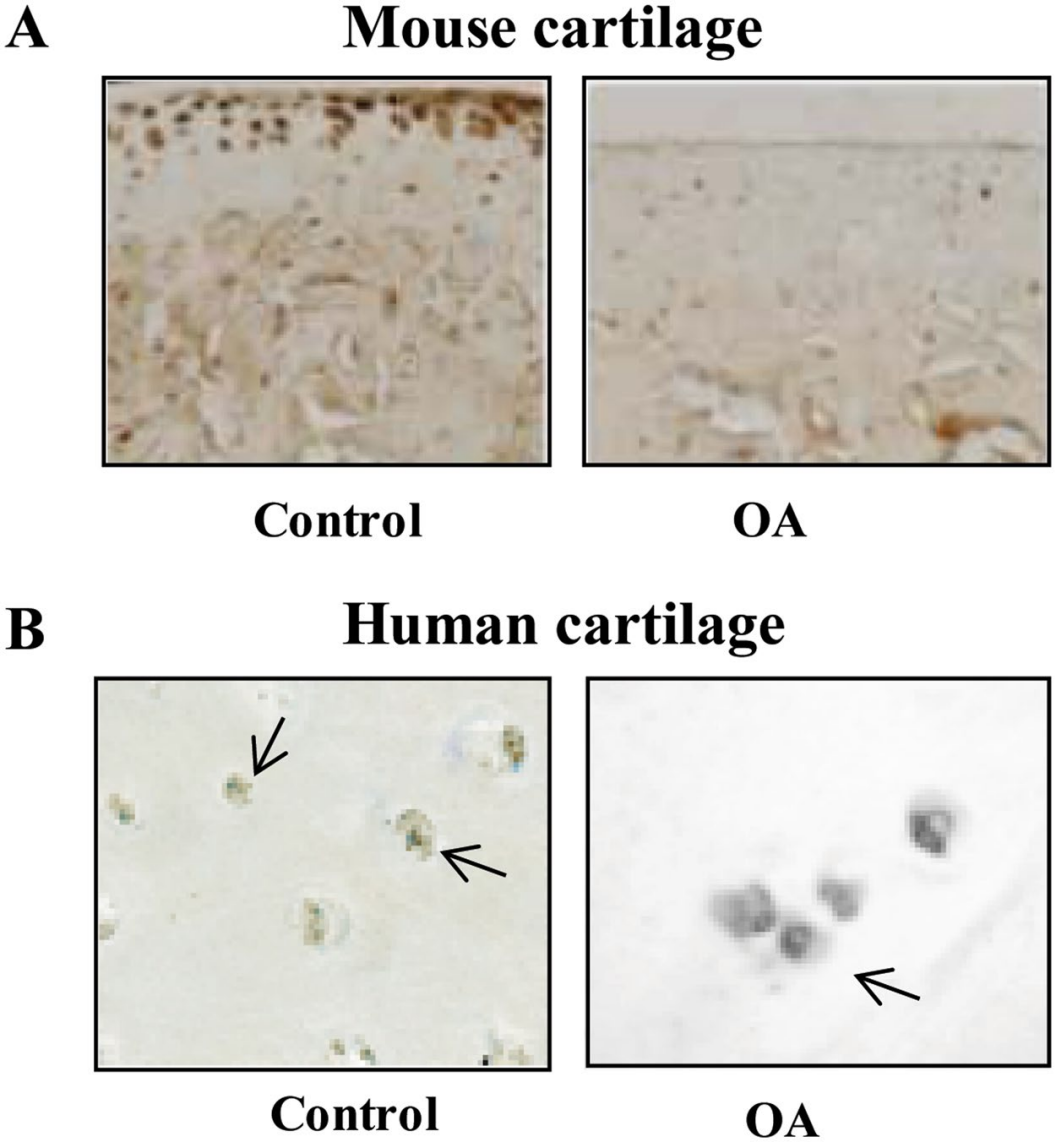
Loss of furin expression in mouse and human OA cartilage. Representative sections of furin assessed by immunohistochemistry in healthy or OA cartilage in mouse (**A**) and human samples (**B**).

We then monitored the expression of furin in OA cartilage in mouse and human cartilage samples. Endogenous furin was expressed in healthy articular cartilage but not in damaged cartilage in mice (Fig. 3A). We further confirmed this finding in human samples (Fig. 3B), suggesting that the loss of furin activity might participate to cartilage degradation in OA. We thus assessed the effect of systemic administration of furin in a mice model of OA. Interestingly, OA score for cartilage lesions was lower with furin than vehicle (6.42 ± 0.75 vs 9.16 ± 0.6, p < 0.01) but not with the furin inhibitor α1-PDX (10.0 ± 0.51, p = NS). By contrast, furin had no impact on subchondral bone or synovium, suggesting that it elicited its effect specifically in cartilage during OA.

In addition, the number of MMP-13(+) cells, which increased significantly in cartilage from OA mice (20.96 ± 8.49% vs control: 2.03 ± 0.19%, p < 0.001, Table 1), was significantly reduced in furin-treated mice (4.96 ± 0.60%, p < 0.05). The proportion of ADAMTS-4(+) cells in cartilage was not significantly affected by Furin (80.06 ± 6.77% vs. control: 86.30 ± 5.22%) (Table 1). α1-PDX did not influence MMP-13 nor ADAMTS-4 expression in OA cartilage.

**Table 1:** Number of protease expressing cells in articular cartilage of mice treated with Furin or antagonist α1-PDX.

	DMM			
	Control	Vehicle	Furin	α1-PDX
MMP-13 (%)	2.03 ± 0.19	20.96 ± 8.49**	4.96 ± 0.60°	23.60 ± 0.14
ADAMTS-4 (%)	10.1 ± 0.25	86.30 ± 5.22**	80.06 ± 6.77	81.06 ± 3.94
ADAMTS-5 (%)	15.8 ± 0.90	62.15 ± 5.86**	57.56 ± 8.98	65.88 ± 6.64

The number of cells expressing MMP-13, ADAMTS-4 or ADAMTS-5 was revealed by immunohistochemistry and expressed as a percentage of total cells.

*vs control: * < 0.05, **p < 0.001; °vs vehicle: °p < 0.05.

**Figure 4:**
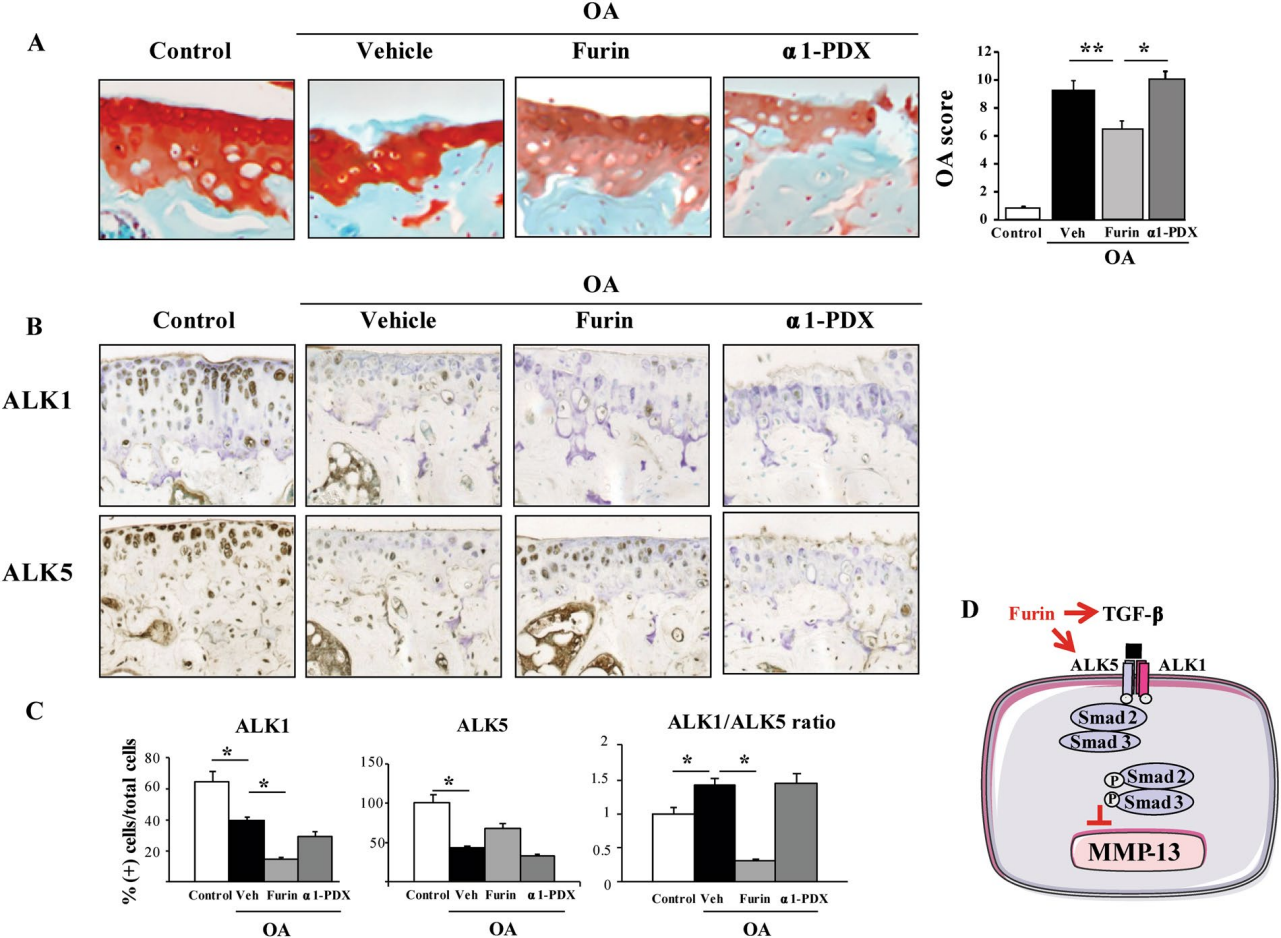
Systemic administration of furin prevents mice against osteoarthritis and regulates ALK1/ALK5 ratio. OA mice were treated with PBS, Furin (1 U/mice) or α1-PDX (14 µg/mice) and compared to sham-operated mice (n = 8 per group). (**A**) Representative sections of joints stained with Safranin-O (X400) and quantification of OA score for cartilage lesions. (**B**) Immunohistochemistry analysis for ALK1 and ALK5 expression (X200). (**C**) quantification of ALK1 and ALK5 (+) cells and ALK1/ALK5 ratio. (**D**) integrative scheme summarizing the effect of furin in chondrocytes. Data are mean ± SEM. *p < 0.05, **p < 0.01.

Immunohistochemistry revealed that OA was associated with decreased number of both ALK5- and ALK1-expressing chondrocytes as compared with controls (48 ± 3.3% vs. 97 ± 9.8% of cells and 40 ± 3.0% vs. 64.7 ± 6.3% of cells, respectively, p < 0.05, Fig. 4B,C). However, ALK1/ALK5 ratio in cartilage was significantly higher in OA vs. healthy cartilage (1.43 ± 0.11 vs 1 ± 0.1, p < 0.05). Furin significantly reduced the number of ALK1 (+) cells but not ALK5 (+) cells (Fig. 3A,B), leading to a reduced ALK1/ALK5 in cartilage from furin-treated mice (0.30 ± 0.22, p < 0.05). This ratio was not affected by α1-PDX treatment (1.45 ± 0.14, p < 0.05). Altogether, these findings suggest that the protective effects of furin on MMP-13 expression in cartilage might be mediated by the modulation of ALK1/ALK5 ratio (Fig. 4D).

## Discussion

Loss of cartilage that characterizes OA occurs mainly by a disrupted balance between the formation and degradation of extracellular matrix as a result of the maturation of growth factors and metalloproteases that are processed by proprotein convertases. Here, we evaluated the role of furin, the main proprotein convertase that metabolizes the activation of these mediators in OA^16,17,19^. We found that systemic treatment with furin alleviated OA cartilage lesions in mice, and that the inhibition of MMP-13 activation is a potential mechanism underlying this chondroprotective effect. Our results also suggest that the inhibition of MMP-13 expression in chondrocytes by furin occurs in an ALK5/Smad3 dependant manner. Indeed, we showed that furin maintains ALK5 and activates Smad3 signaling. This is consistent with previous study reporting that the TGFβ signaling regulates MMP-13 expression^10^ and that Smad3 can repress MMP-13 expression in chondrocytes^20^.

The age-related increase in ALK1/ALK5 ratio observed in OA cartilage occurs mostly through a down-regulation of ALK5^14,21^. We show here that the inhibition of MMP-13 expression induced by furin was reversed by ALK5 knockdown in IL-1-stimulated chondrocytes. This suggests that furin inhibits MMP-13 expression by upregulating ALK5, in line with previous evidence that MMP-13 expression is increased after ALK5 knockdown in mice^15^. Consistently, furin maintained the expression of ALK5 in cartilage in our experimental model of OA, thus preventing the activation of MMP-13 and allowing the preservation of cartilage remodeling. These results are consistent with previous findings that deletion of Smad3 results in an OA phenotype in mice^13^. The shift from ALK5 to ALK1 signaling occurring with age is an important mechanism of cartilage damage in OA^22^. Here, furin maintained the levels of ALK5 and prevented the increased ALK1/ALK5 ratio in OA mice at the cell and tissue levels. Further evidence was provided by the furin inhibitor α1-PDX, which reversed the phenotype, and even promoted the expression of MMP-13 and aggrecanases. This supports a physiological role for endogenous furin to regulate cartilage catabolism and thus to limit post-traumatic OA in mouse knees by maintaining ALK5 and downregulating MMP-13. Consistently, we observed that furin was expressed in undamaged cartilage of humans and mouse while lost in OA cartilage, indicating that furin is involved in the protection of cartilage damage. However, the loss of furin expression in OA cartilage also suggest that this physiological mechanism is insufficient to prevent OA by itself, and supports the pharmacological relevance of the systemic administration of exogenous furin in OA. However, we cannot rule out an effect on other tissues because the administration was systemic but not local. The ability of furin to reduce MMP-13 expression in primary chondrocytes is mainly linked to furin-induced TGF processing and activity as a downstream effector.

Here, the administration of furin did not diminish the expression of aggrecanases in OA cartilage or in IL-1-treated chondrocytes. Despite the essential function of furin for cleavage of several proteases in chondrocytes and cartilage explants^19,23–26^, furin might promote the processing of extracellular and intracellular proteins by several mechanisms^25,27^. Furin could have regulated the activation of ADAMTS in the matrix and facilitated the cartilage degradation by posttranscriptional processes, without affecting cellular expression^26^.

As discussed, our findings suggest that α1-PDX acted also on the endogenous Furin, without promoting Smad3 translocation or affecting ALK1/ALK5 ratio. The absence of regulation of TGFβ receptors by α1-PDX in primary chondrocytes suggests additional mechanisms induced by α1-PDX, a nonspecific inhibitor that might regulate several other proprotein convertases such as PACE4^24,26,28^. In addition to the direct effects in chondrocytes, systemic administration could have targeted other joint tissues, such as the synovium. Indeed, we have previously shown that low-grade synovitis was a common feature in the post-traumatic model of OA^29^. We have also previously demonstrated that α1-PDX was able to inhibit the regulatory T cells and promote synovitis and joint damage in a mice model of collagen-induced arthritis^30^. Therefore, α1-PDX could have also promoted some level of inflammatory response that contributed to cartilage damage even in the OA mechanical model.

Altogether, these data demonstrate that systemic administration of furin negatively regulates MMP-13 by maintaining ALK5 receptors and Smad3 signaling in OA, thus reducing cartilage degradation in mice. We here highlight the potential use of furin or other agonists for treatment of cartilage loss.

## Methods

### Chondrocyte cultures

Primary cultures of murine articular chondrocytes were obtained from newborn mice as previously described^31^. Briefly, joint cartilage was isolated from femur of 6-day-old mice. To separate chondrocytes from matrix, cartilage underwent 3 successive digestions: the first 2 digestions involved incubating the samples with liberase (Roche, Switzerland), 0.52 U/mL, for 45 min at 37 °C, then overnight with 0.13 U/mL liberase. The solution was passed through 25-, 10-, 5-, 2-ml Pasteur pipettes successively to break any aggregates. Then, the isolated cell suspension was filtered through a sterile 100-μM cell strainer and centrifuged at 2000 g for 15 min. The pellet was suspended in 3 mL DMEM with 10% fetal bovine serum (FBS) and seeded at 40000 cells per cm^2^. Confluence was reached after 6 days of culture. Chondrocytes were pre-stimulated with interleukin 1 (IL-1) at 10 ng/mL for 24 h, washed and then cultured for 96 h with PBS, or with Furin 10 U/mL (P8077L, New England Biolabs); or with Furin inhibitor α1-PDX, 8 µM (RP-070 Thermo Scientific); and/or the TGFβ inhibitor SB431542, 50 µM (S4317, Sigma Aldrich France). Control chondrocytes were cultured in the presence of phosphate buffered saline (PBS) alone after seeding.

### RT-PCR

RNA extraction involved use of the RNeasy mini kit (74126 Qiagen). Total RNA was converted to cDNA by use of the cDNA verso Kit (Thermo Fisher Scientific, UK). Relative mRNA levels were evaluated by quantitative PCR with LightCycler (Roche Applied Science, Switzerland) in Absolute Blue qPCR SYBR Green (Thermo Fisher Scientific), at fusion temperature 60 °C, with 40 cycles, with the following primers: ADAMTS4: 5′TCAGCCCAAGGTGAGTG3′ and 5′GGCAAGGACTATGACGC3′; ADAMTS5: 5′TCAGCCACCATCACAGAA3′ and 5′CCAGGGCACACCGAGTA3′; MMP13: 5′TGTAGCCTTTGGAACTGCTT3′ and 5′TGATGGCACTGCTGACATCAT3′; Aggrecanase: 5′CAGGGTTCCCAGTGTTCAGT3′ and 5′CTGCTCCCAGTCTCAACTCC3′; collagen type II (COL2A1): 5′CCGTCATCGAGTACCGATCA3′ and 5′CAGGTCAGGTCAGCCATTCA3′); ALK1: 5′TGGCAGCTGTCGATATAGCAT3′ and 5′ACACAACGGACAAATCGTCTAC; ALK5: 5′AGAGGTGGCAGAAACACTGTAAT3′ and 5′GCAGCTCCTCATCGTGTTG3′; RPL13A: 5′GCCGAACAACCTTGAGAGC3′ and 5′GGATCCCTCCACCCTATGACA3′ for normalisation. Results are expressed in fold increase in ΔΔCT normalised for the control conditions.

### SiRNA knock-down

SiRNA interference was performed using siRNA against ALK5, or control siRNA (Santa Cruz Biotechnology, CA). For this assay, primary murine chondrocytes were cultured at 70% confluence, and transfected with Exgen 500 (Euromedex, Strasbourg, France) with 1 µg siRNA according to the manufacturer’s instructions. After 24 hours, the incubation medium was removed and replaced with fresh medium with different treatments. 24 hours after, total RNA was then collected for quantitative real-time PCR analysis. Control of transfection efficiency was checked also by quantitative real-time PCR.

### Western blot analysis

After stimulation with IL-1 (10 ng/mL) for 24 h, cell extracts from primary chondrocytes incubated with Furin (10 U/mL), α1-PDX (8 µM) and/or SB431542 (50 µM) were prepared by adding 1 mL of ice-cold lysis buffer containing 10 mM Tris–HCl, 5 mM EDTA, 150 mM NaCl, 30 mM sodium pyrophosphate, 50 mM NaF, 1 mM Na3VO4, 10% glycerol, and protease inhibitors (Boehringer, Mannheim, Germany). Western blot analysis was performed as previously described^32^. We used rabbit polyclonal antibodies against MMP-13 (ab39012 Abcam, UK), ß-actin (Santa Cruz Biotechnology, USA), p-Smad2, p-Smad3, Smad2/3 (Cell Signaling, France) and horseradish peroxidase (HRP)-conjugated secondary antibody. Signals were detected by use of the Immun-Star Western C kit (170–5070 Biorad, France) and chemiluminescent kit (Amersham) with a FujiFilm LAS-3000 Intelligent dark box (Fuji, France). Optical density of bands was assessed with Image J. Membranes for western blot analysis were also tested for Actin for quantification of protein/actin signals. Results are expressed as change in expression relative to controls.

### Immunofluorescence analysis

Primary chondrocytes cultured on sterile coverslips were activated with IL-1, 10 ng/mL, then stimulated overnight with furin (10 U/mL), α1-PDX (8 µM), SB431542 (50 µM) and/or TGFβ (0.02 ng/mL; 243-B3 R&D Systems). Chondrocytes were fixed with 4% paraformaldehyde and incubated with polyclonal Smad2/3 antibody (5678 S, Cell Signaling, France), then Cy2-conjugated immunoglobulin (Jackson Immunoresearch, West Grove, PA). Nuclei were stained with DAPI. Images were acquired with Zeiss Apotome and Axiovision software (Carl Zeiss, Jena, Germany) with a 63×/1.4 objective lens.

### Animals

The experiments complied with the guidelines for animal experimentation issued by the local ethics committee on animal care and experimentation and approved (Ethical committee Lariboisière-Villemin, IRB n° 0000383, Paris). To induce joint instability of the knee, 10-week-old male C57/Bl6 mice (Janvier, France) underwent destabilization of the medial meniscus (DMM) of the right knee as previously described^33^. The left knee underwent sham surgery and used as control. After DMM, mice were divided into 3 groups (n = 8 mice per group) and received 3 times a week for 6 weeks intraperitoneal injections of 1) Furin (1 U/mice), 2) Furin inhibitor α1-protease inhibitor Portland (α1-PDX) (14 µg/mice) or 3) phosphate buffered saline (vehicle). We purchased Furin (ref P8077L, New England Biolabs) and α1-PDX (ref RP-070, Thermo Scientific ABR).

The total amounts Furin and α1-PDX were provided in the same bulk by the manufacturer. Doses and schedule were determined in previous experiments and shown to be efficient for preventing arthritis in a collagen induced model^34^. In these conditions, mice did not show any abnormality in terms of growth, weight and behavior. At sacrifice, no macroscopic lesions were observed in any tissues examined.

### Preparation of mouse and human joint samples

After sacrifice of the mice, whole knee joints were dissected free of soft tissues (n = 8 per group). All specimens were fixed with 4% paraformaldehyde (PFA; pH 7.4) for 24 h at 4 °C, and then decalcified with 1% PFA-0.2 M EDTA (pH 7.4, 4 °C) for 2 weeks, with the solution changed twice a week. The specimens were dehydrated with increasing concentrations of ethanol before being embedded in paraffin. Serial 5 µm thick sagittal sections were cut in the medial femoro-tibial compartment for histology and immunohistochemistry as described^35^.

Human cartilage samples were harvested from three patients who underwent total knee replacement surgery. Samples were obtained in accordance with the guidelines and regulations of the French National Authority Legislation for the collection of human tissues. Collections were approved by the ethical committee of the institution, informed consents were obtained from patients and stored in the medical record. Cartilage samples were collected from the femoral condyle at the posterior surface of the knees. We collected samples in a zone that appeared macroscopically undamaged defined by white and shiny cartilage without lesions and in a zone that appeared damaged defined by a discoloration with an irregular surface. All samples were fixed in 4% PFA, processed as described above and embedded in paraffin until processed for immunohistochemistry for Furin.

### Histology and immunohistochemistry

Human cartilage samples were harvested from three patients who underwent total knee replacement surgery, in accordance with the French National Authority Legislation for the collection of human tissues. Cartilage samples were collected from the femoral condyle at the posterior surface of the knees. For each patient, we collected samples in 2 different zones: one sample in a zone that appeared macroscopically undamaged defined by white and shiny cartilage without lesions and one sample in a zone that appeared damaged defined by a discoloration with an irregular surface. All samples were fixed in 4% PFA and embedded in paraffin until processed for immunohistochemistry for Furin.

The blocks of the 4 groups of mice were processed (n = 8 per group). Serial 5 µm thick sagittal sections were cut, deparaffinized, rehydrated and washed twice by 5-min immersions in baths of distilled water. Sections underwent Mayer-Hemalun staining for 5 min to stain the nuclei, and then counterstained with 0.125% Fast Green for 2 min for bone tissue. OA lesions were assessed in sections stained with Safranin-O. Sections were stained with 0.5% Safranin-O for quantification of OA scores^36^. Osteoarthritic lesions were scored by the OA scale ranging from stage 0 (normal) to 6 (vertical clefts/erosion to the calcified cartilage extending over >75% of the joint surface) in the medial compartment of tibia and femoral articular cartilage, for a global score of 0 to 12.

Immunohistochemistry was performed on serial sections as previously described^33^ by use of the Vector kit (PK-6101, Abcys, France). Sections were incubated with the primary polyclonal antibodies for Furin (sc-20801, Santa Cruz Biotechnology, France) as well as the expression of ADAMTS using the following antibodies (murine ADAMTS-4, ab28285), ADAMTS-5, ab41037, Abcam, UK for both) and MMP-13 (ab3208, Abcam, France). Sections were counterstained with Toluidine blue. Slides with no primary antibody added were used as controls. Positive cells were counted on the articular cartilage of the tibia (80X magnification). We counted the number of cells expressing these metalloproteases and the number of total cells. Results are expressed as a percentage of positive cells to total cells.

The thickness of the synovium was measured in the serial sections stained for SO in the 4 groups of mice (n = 8). Synovial thickness was measured using a semi-automatic method with an image analyzer (Microvision, France). Data are expressed as mean ± SEM.

All the above experiments methods were carried out with the relevant guidelines and regulations and the protocols approved by the local scientific committee of the institution (Lariboisière-Villemin, IRB n° 0000383, Paris).

### Statistical analysis

Results are expressed as mean ± SEM of at least 3 separate experiments. The effect of DMM on mice was analysed by ANOVA followed by Wilcoxon rank or Mann-Whitney tests as appropriate. The latter test was also used to examine results of chondrocyte culture conditions. Level of significance was set at p < 0.05. Statistical analyses were performed using Statview software (SAS Inst., Cary, NC USA).
